# Robust Self-Composite Polyimide Separators for Long-Life Lithium-Ion Batteries

**DOI:** 10.1021/acsomega.6c00560

**Published:** 2026-04-14

**Authors:** Shilong Bai, Ping Gao

**Affiliations:** † Advanced Materials Thrust, Function Hub, The Hong Kong University of Science and Technology (Guangzhou), Nansha District, Guangzhou, 511453, China; ‡ Department of Chemical and Biological Engineering, 567841 The Hong Kong University of Science and Technology, Clear Water Bay, Hong Kong SAR, P.R. China

## Abstract

Electrospun polyimide (PI) nanofiber separators hold promise for high-performance lithium-ion batteries due to their exceptional thermal stability and affinity for electrolytes. However, their practical application is limited by inadequate mechanical integrity and large, nonuniform pores that fail to suppress lithium dendrite growth. To address these challenges, we developed a “self-composite” strategy that integrates electrospinning with non-solvent-induced phase separation (NIPS). This method produces a hierarchical self-composite polyimide (SCPI) separator, featuring a robust electrospun PI nanofiber scaffold infilled with and bonded by a NIPS-derived PI matrix. The resulting monolithic composite exhibits a 230% increase in tensile strength over a pristine electrospun membrane and a significant decrease in average pore size from the micrometer scale (∼1–5 μm) down to 200–500 nm. This uniform nanoporous structure and superior wettability effectively homogenize lithium-ion flux, enabling dendrite-free lithium plating/stripping even at a high current density of 5 mA/cm^2^. Consequently, Li/LFP pouch cells with SCPI separators achieve outstanding long-term stability, retaining 98.75% of their initial capacity after 800 cycles at 1 C with a Coulombic efficiency exceeding 99.99%. This scalable approach offers an effective pathway to engineer advanced membrane structures for safe, high-performance, next-generation lithium batteries.

## Graphical abstract

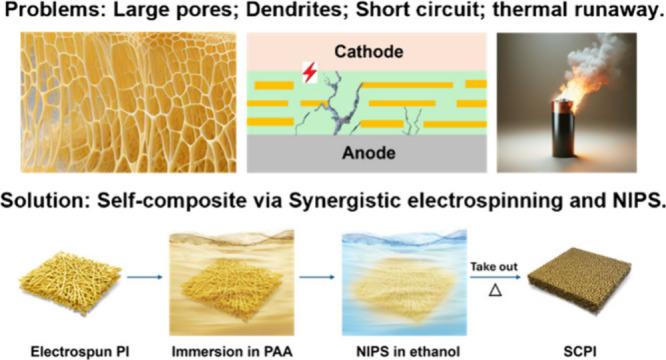

## 1 Introduction

Rechargeable lithium-ion batteries (LIBs) have become indispensable over the past two decades, powering diverse applications from portable electronics to electric vehicles and grid-scale energy storage.^1,2^ Their success stems from high specific energy density, minimal memory effect, and extended cycle life.^2,3^ A typical LIB comprises an anode, cathode, electrolyte, and separator. Although the separator does not participate directly in electrochemical reactions, it plays a vital safety role by preventing electrical shorting while facilitating efficient lithium-ion transport.^4–10^

Despite well-known advantages, conventional polyolefin separators (e.g., polyethylene or polypropylene) exhibit significant limitations, especially in next-generation high-energy-density batteries.^4–7^ These materials suffer from significant drawbacks, including poor thermal stability and pronounced thermal shrinkage at elevated temperatures, which can lead to internal short circuits and catastrophic thermal runaway.^8–12^ Additionally, their inherent hydrophobicity impairs wettability with polar electrolytes, increasing internal resistance and reducing ionic conductivity—ultimately compromising battery performance.^13–15^ As the battery industry advances toward lithium metal anodes and high-voltage systems, there is an urgent need for separators that can suppress dendrite growth and endure a harsh electrochemical environment.^5,16–19^

To overcome these shortcomings, researchers have explored polymers with inherent thermal stability and compatible surface chemistry. Polyimide (PI) stands out as a prime candidate, boasting decomposition temperatures above 500 °C, robust mechanical properties, excellent electrochemical stability, and strong wettability with organic electrolytes due to its polar imide groups.^20,21^ Electrospinning is a widely used technique to fabricate porous PI membranes from a polyamic acid (PAA) precursor, followed by thermal imidization.^22^ However, pristine electrospun PI separators are hampered by two major issues that limit their adoption: large, irregular pores (often several microns in diameter) that fail to prevent micro-short circuits or regulate uniform ion flux for dendrite suppression, and low tensile strength (<10 MPa) resulting from weak interfiber bonding, which leads to handling difficulties during assembly.^23^ Non-solvent-induced phase separation (NIPS) is another approach to prepare polyimide separators. But the porosity of NIPS-based separators is typically around 70%, which is relatively lower than that of electrospun PI separators. Furthermore, controlling the formation process and size distribution of pores via NIPS is challenging because it is hard to precisely control the diffusion rate of two solvents, leading to the appearance of figure-like structures and macro-voids.^24^

Building on these insights, we present an innovative strategy to resolve these trade-offs via the development of a self-composite polyimide (SCPI) separator. Our method combines electrospinning and NIPS in a two-stage process, as illustrated in Figure 1b. Initially, an electrospun PI nanofiber mat forms a structural scaffold, which is then impregnated with a PAA solution. NIPS precipitates a PAA matrix within the scaffold’s voids, and final thermal imidization integrates the structure. This yields a monolithic composite from a single material, avoiding interfacial incompatibility. The resulting hierarchical architecture features reduced pore sizes, markedly enhanced mechanical strength, and improved electrochemical performance. Critically, the uniform nanoporous network suppresses lithium dendrite growth, enabling superior cycling stability in practical pouch cells and underscoring its promise for advanced energy storage systems.

**Figure 1:**
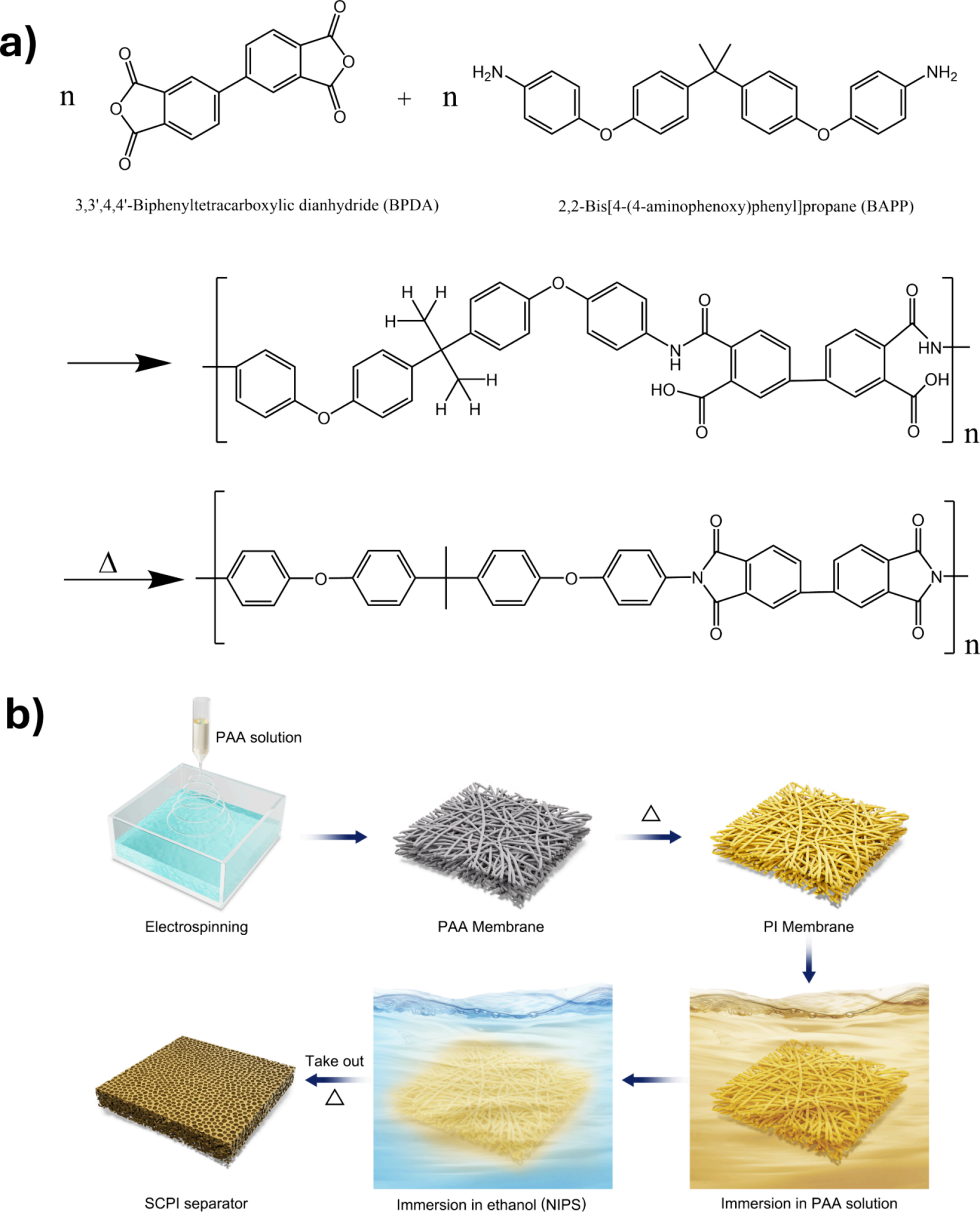
(a) Two-step synthesis of the polyimide (PI) from BPDA and BAPP monomers via a polyamic acid (PAA) precursor. (b) Schematic illustration of the fabrication process for the self-composite polyimide (SCPI) separator, synergistically combining electrospinning and non-solvent-induced phase separation (NIPS).

## 2 Experimental Section

### 2.1 Chemicals

Two monomers, 3,3′,4,4′-biphenyltetracarboxylic dianhydride (BPDA) and 2,2-bis­[4-(4-aminophenoxy)­phenyl]­propane (BAPP), and *N*,*N*-dimethylacetamide (DMAc), the solvent, were purchased from Shanghai Aladdin Biochemical Technology Co. Ltd.

### 2.2 Polyamic Acid (PAA) Synthesis

BPDA and DMAc were added into a three-necked flask equipped with a mechanical stirrer. Subsequently, a solution of BAPP in DMAc was added dropwise to the flask via an injection pump, while the flask was maintained in an ice-bath. The molar ratio of BPDA to BAPP was 1:1. The reaction was allowed to proceed until the Weissenberg effect was observed, indicating a high molecular weight. This process yielded a 15 wt % PAA solution.

### 2.3 Preparation of Electrospun Polyimide Membranes

The PAA solution was electrospun by using an electrospinning instrument (MECC, Nano-01B) at a voltage of 20.0 kV and an injection speed of 0.2 mL/h. A smooth water surface was employed as the collection medium, which facilitates rapid solvent removal and nanofiber solidification, potentially leading to smaller fiber diameters. The resulting electrospun PAA membranes were transferred onto poly­(tetrafluoroethylene) (PTFE) substrates and heated at 260 °C for 1 h for imidization.

### 2.4 Preparation of Self-Composite Polyimide Separators

The self-composite polyimide (SCPI) separators were prepared by a two-step process: non-solvent-induced phase separation (NIPS) followed by a solid-state imidization. Briefly, the electrospun PI membranes were dipped into a 7 wt % PAA solution for ∼1 h. The fully saturated PI membranes containing a PAA solution were transferred into pure ethanol for 30 min to induce phase separation. Upon drying, the non-solvent-induced PAA membranes were subjected to a second-step thermal imidization process: 100 °C for 1 h, 150 °C for 1 h, 200 °C for 1 h, and 260 °C for 1 h to yield the final SCPI separators. For this preparation process, the 7 wt % PAA solution was prepared by directly diluting the original 15 wt % PAA solution using DMAc. The concentration of the PAA solution has a profound influence on the pore-filling and phase-inversion dynamics. When the concentration of the PAA solution exceeds 10 wt %, the viscosity becomes too high; this severely hinders the phase separation process, resulting in a dense layer where no new pores can form on the membrane surface. Through our optimization, 7 wt % was found to be the ideal concentration. At this concentration, the viscosity is appropriate to allow the PAA to uniformly coat the electrospun framework and successfully generate properly sized, interconnected pores during the NIPS process.

### 2.5 Characterization of Separators

The chemical structure of the synthetic polyimide was confirmed by a Fourier transform infrared spectrometer (Bruker, Vertex 70v) in the transmission mode from 4000 to 400 cm^–1^. The polyimide’s thermal stability was evaluated by a thermogravimetric analyzer (TA, TGA 5500). The morphologies of separators and lithium metal electrodes were observed using scanning electron microscopy (SEM) (Hitachi, Regulus 8230 and 8100). The size distribution of pores was characterized by a porosimeter (Poretech, Innova Porometer iUNP-1500AEX).

Separator porosity was estimated by immersing films in propylene carbonate (PC) for 72 h. After excess surface solvent was removed, the mass of the dry (*m*_df_) and wet films was recorded. Porosity was calculated using eq 1, where ρ_pc_ and ρ_pi_ are the densities of PC and polyimide, respectively. Electrolyte uptake was calculated using eq 2.

$$\mathrm{Porosity}=\left(m_{\mathrm{pc}}/\rho_{\mathrm{pc}}\right)/\left(m_{\mathrm{pc}}/\rho_{\mathrm{pc}}+m_{\mathrm{df}}/\rho_{\mathrm{pi}}\right)\times 100\%$$ 1

$$\mathrm{Electrolyte}\;\mathrm{Uptake}=m_{\mathrm{pc}}/m_{\mathrm{df}}\times 100\%$$ 2

The tensile strength of separators was tested by an electromechanical universal testing machine (Instron, 68TM-5). Wettability toward water and PC was evaluated using a contact angle meter (Biolin, Theta Flow).

### 2.6 Electrochemical Tests

Ionic conductivity was determined from the electrochemical impedance spectra (EIS) of stainless steel (SS)/separator/SS symmetric cells. EIS was recorded on a Gamry electrochemical workstation (5 mV amplitude, 1.0 MHz to 0.01 Hz). The electrochemical stability window was evaluated by linear scanning voltammetry (LSV) of Li/separator/SS cells from open circuit voltage to 6.0 V vs Li/Li^+^ at 5 mV/s. To measure the Li^+^ transference number, Li/Li symmetric cells were assembled and potentiostatic polarization at 10 mV was applied for 6000 s by a Gamry electrochemical workstation. The impedance spectra of the cells before and after polarization were recorded at 10 mV, from 1.0 MHz to 0.1 Hz. The Li^+^ transference number was calculated using eq 3, where Δ*V* is the bias voltage, *I*_0_ and *I*_s_ are the initial and steady state currents, and *R*_0_ and *R*_s_ are the electrochemical resistances of the cell before and after the polarization, respectively. For these tests, the electrolyte was 1 M lithium bis­(trifluoromethanesulfonyl)­imide (LiTFSi) in a 1:1 (v/v) mixture of ethylene glycol dimethyl ether (DME) and 1,3-dioxolane (DOL).

$${\mathrm{Li}}^{+}\;\mathrm{transference}\;\mathrm{number}=\left(I_{s}/I_{0}\right)\times \left(\Delta V-I_{0}R_{0}\right)/\left(\Delta V-I_{s}R_{s}\right)$$ 3

Li/Li symmetric cells were assembled with the same electrolyte and cycled galvanostatically at 5 mA/cm^2^ with a capacity of 5 mAh/cm^2^. After 100 cycles, the cells were disassembled in an argon-filled glovebox for the SEM analysis of the lithium electrodes. Rate capability and cycling performance were examined in Li/lithium iron phosphate (LFP) cells using 1 M LiTFSi in DME/DOL (1:1 v/v) with 1% LiNO_3_ additive. For practical assessment, full pouch cells were assembled with graphite anodes, LFP cathodes, and a commercial electrolyte (TC-EGKD001, Jiujiang Tianci Materials Co. Ltd.). The pouch cells were tested at a 1 C rate. All cells were assembled in an argon-filled glovebox.

## 3 Results and Discussion

### 3.1 Synthesis and Structural Characterization of Polyimide

The polyimide was synthesized from BPDA and BAPP monomers via a two-step polycondensation and imidization process (Figure 1a). The flexible ether linkages and bulky C­(CH_3_)_2_ groups in the BAPP monomer were intentionally chosen to enhance the solubility of PAA in DMAc, facilitating the electrospinning process. The chemical structure of the final PI material was confirmed by FT-IR spectroscopy (Figure 2a). The characteristic absorption peaks for the imide ring are clearly visible at 1775 cm^–1^ (asymmetric C=O stretching) and 1721 cm^–1^ (symmetric C=O stretching), along with the C–N stretching vibration at 1376 cm^–1^. The disappearance of broad peaks associated with amic acid (−COOH and −NH) confirms successful and complete imidization. The resulting thermal stability of the fully imidized PI was evaluated by TGA (Figure 2b), which shows a high decomposition temperature (*T*_d_, 5% weight loss) of approximately 518 °C, confirming the material’s suitability for high-safety battery applications.

**Figure 2:**
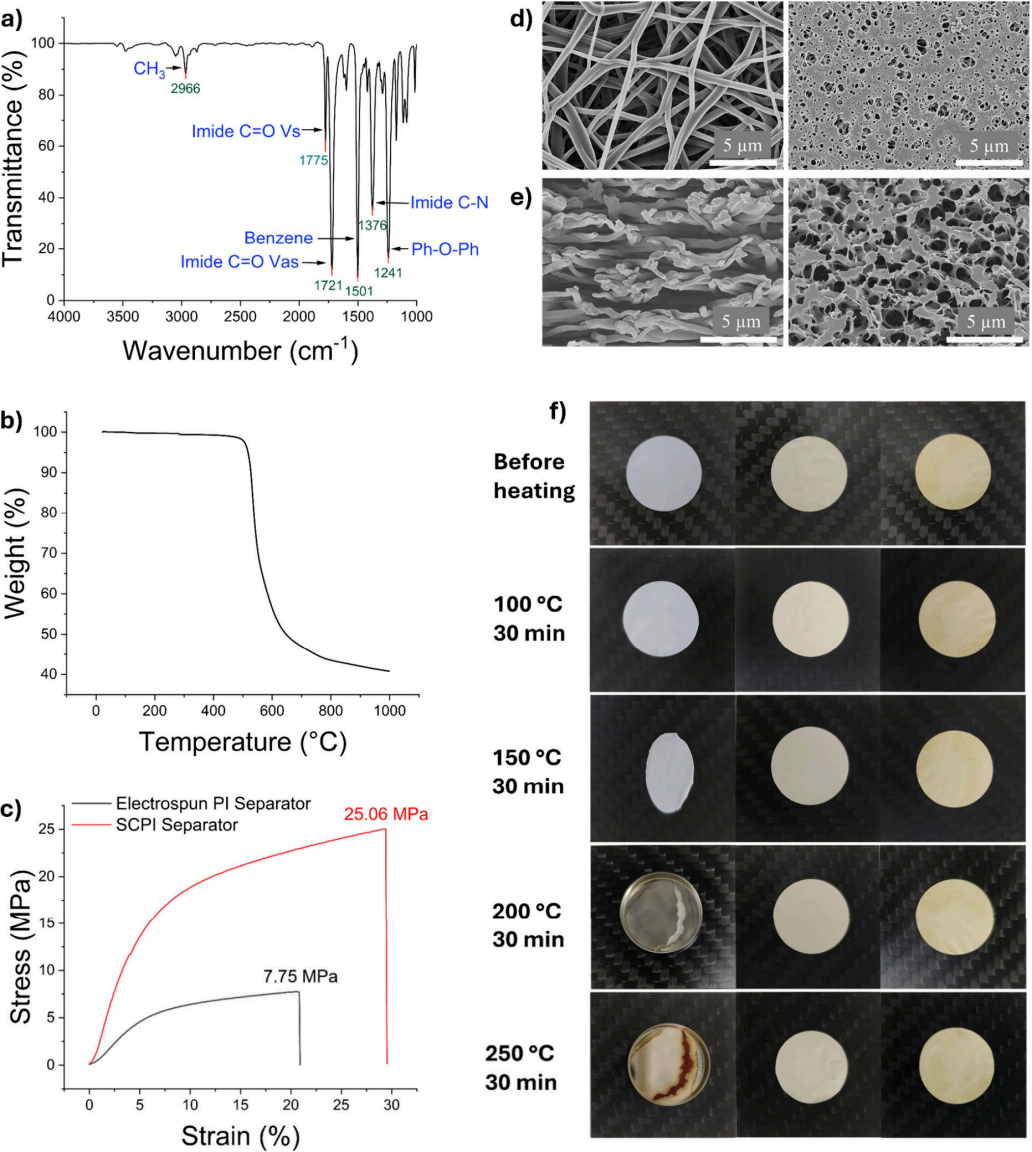
a) FT-IR spectrum of the as-synthesized self-composite polyimide. b) TGA of the polyimide. c) Stress–strain curves measured at an ambient temperature at a constant strain rate of 10 mm/min on a universal tensile tester. d,e) SEM images of morphologies. Left: Electrospun PI separators; right: SCPI separators. Scale bar: 5 μm. d: surface; e: cross-section. f) Dimensional stability tests of the separators at elevated temperatures. In every subgraph, left: Celgard2500 PP; middle: electrospun PI; right: SCPI. The backing for each of the separators was square carbon boards with dimensions of 37 × 37 mm^2^.

### 3.2 Morphological Evolution and Hierarchical Structure of the SCPI Separator

The key to our work lies in the morphological transformation from a standard electrospun PI mat to the robust SCPI separator, a process detailed in Figure 1b. Figures 2d,e and Figure S1 present a direct comparison of the surface and cross-sectional morphologies. The pristine electrospun PI separator (Figure 2d, left) exhibits a typical nonwoven structure of randomly overlaid nanofibers, creating large, irregular polygonal pores with sizes ranging from 1 to 5 μm. The cross-section (Figure 2e, left) reveals significant void space and large transverse distances between fibers, which contribute to its poor mechanical integrity.

In stark contrast, the SCPI separator (Figure 2d, right) displays a dramatically different surface morphology. The original large pores are filled, replaced by a continuous matrix featuring much smaller and more uniform circular pores with diameters of 200–500 nm. The cross-sectional view (Figure 2e, right) reveals the underlying mechanism: the NIPS-derived polyimide has filled the large interfiber voids, effectively “gluing” the nanofiber scaffold together. This creates a hierarchical, integrated pore-in-pore structure, which is key to the separator’s enhanced properties.

This significant reduction in the pore size was quantitatively confirmed by porosimetry measurements (Figure S2). While the electrospun PI separator shows a broad pore size distribution peaking above 600 nm and extending to nearly 2 μm, the SCPI separator exhibits a much narrower distribution, with the vast majority of pores concentrated between 100 and 800 nm. This engineered pore structure is expected to play a crucial role in regulating ion transport and influencing electrochemical performance.

### 3.3 Mechanical, Thermal, and Wetting Properties

The structural reinforcement from the NIPS-derived matrix profoundly affects the separator’s mechanical properties. As shown in the stress–strain curves in Figure 2c, the pristine electrospun PI separator exhibits a low tensile strength, consistent with literature values below 10 MPa,^25,26^ which is insufficient for robust battery manufacturing, requiring a minimum strength of 13 MPa.^27^ Our SCPI separator demonstrates a tensile strength approximately three times higher, comfortably exceeding this industrial benchmark. This 230% enhancement is directly attributed to the NIPS-derived PI acting as a binder, creating stronger adhesion points between nanofibers (Figure 2e, right) and reducing the overall porosity (Table 1), consistent with scaling laws for porous materials.

**Table 1:** Electrolyte Uptakes and Porosities of Different Separators

	Average	
Separator Type	Electrolyte Uptake (%)	Porosity (%)
Celgard2500 PP separators	169	55.9
Electrospun PI separators	670	87.6
SCPI separators	420	81.3

The thermal stability of the separators is critical for battery safety. Figure 2f displays the dimensional stability of the separators following a 30 min thermal treatment at elevated temperatures. The commercial PP separator undergoes severe shrinkage at 150 °C and completely melts at 200 °C. In contrast, both the electrospun PI and SCPI separators, benefiting from the intrinsic stability of polyimide, show no visible shrinkage or dimensional change even at 250 °C, which demonstrates their outstanding advantage for preventing thermal runaway.

The electrolyte uptake of separators significantly influences their electrochemical properties. The electrolyte uptakes of the three types of separators are listed in Table 1. Both electrospun PI and SCPI separators have much higher electrolyte uptakes and porosities than commercial Celgard2500 PP separators. But the porosity of SCPI separators is slightly lower than that of electrospun PI separators, indicating that part of the space in the original pores has been occupied and the big pores have been separated into many small pores. This result aligns with the conclusions drawn from the SEM and porometer tests.

Excellent electrolyte wettability is essential for low interfacial resistance and efficient ion transport. As shown in Figures 3a,b and S3, the nonpolar PP separator exhibits poor wettability, maintaining a high contact angle of 74° with the polar PC electrolyte. Conversely, both PI-based separators show superoleophilic behavior, with the PC droplet being completely absorbed in under 1 s, resulting in an effective contact angle of 0°. Interestingly, all separators express hydrophobic responses to water. This dual characteristic is highly desirable: hydrophobicity simplifies handling in controlled-humidity environments during battery assembly, while the exceptional electrolyte affinity ensures rapid and thorough wetting, reducing cell impedance and conditioning time.

**Figure 3:**
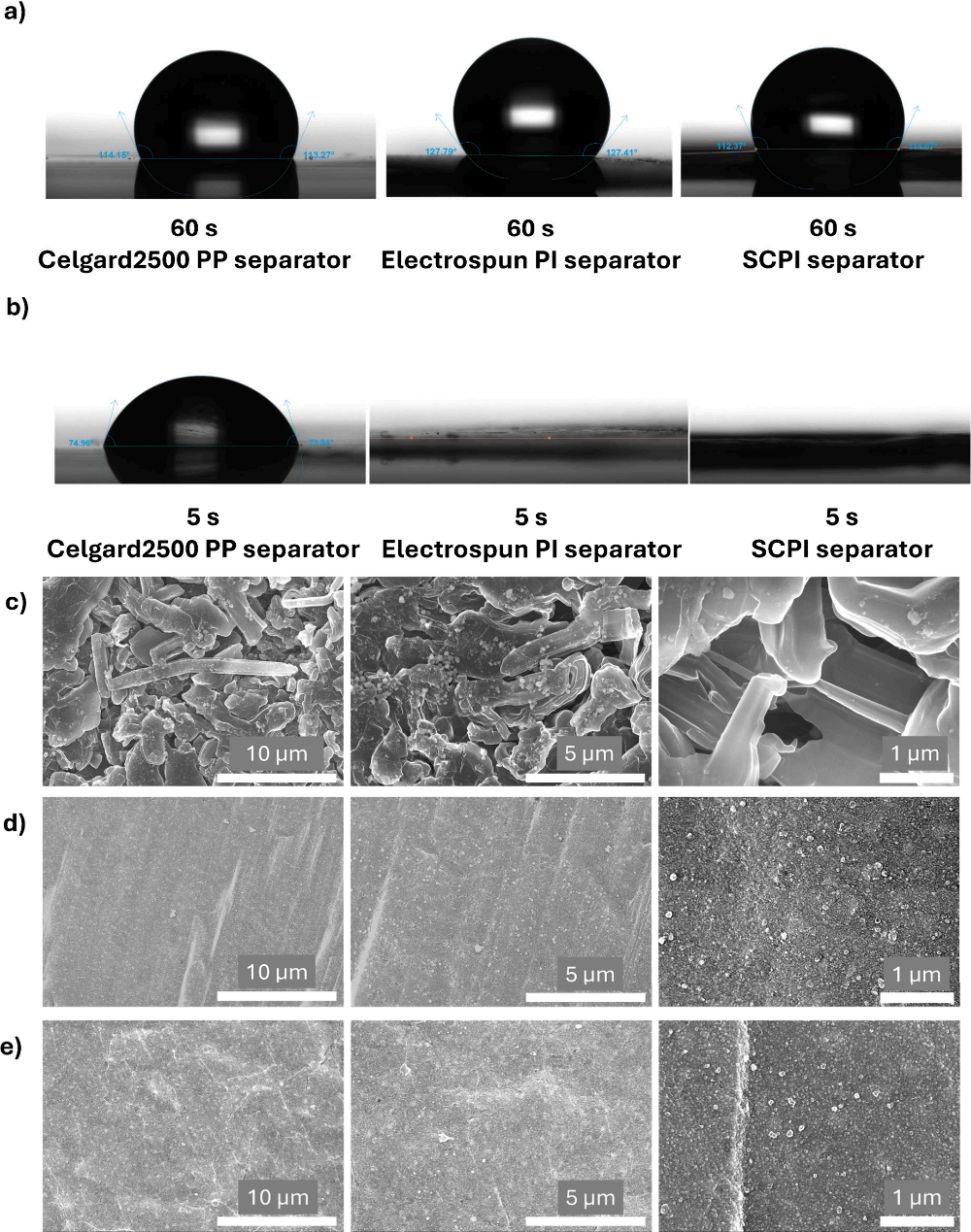
a,b) Contact angles on the three types of separators. a: Water. b: PC. c–e) Morphologies of lithium metal electrodes in the Li/separator/Li symmetric cells equipped with different separators. The images were displayed at increasing magnifications from left to right. Scale bar: 10 μm (left); 5 μm (middle); 1 μm (right). c: Celgard2500 PP; d: electrospun PI; e: SCPI.

### 3.4 Electrochemical Performance and Dendrite Suppression

The SCPI separator’s hierarchical structure yields superior electrochemical performance. Its ionic conductivity of 1.59 mS/cm (Figure 4c) surpasses that of the PP separator (1.34 mS/cm), although it remains lower than that of the electrospun PI separator (3.59 mS/cm). The decrease in the ionic conductivity from electrospun PI to SCPI indicates a tortuosity increase. This change in tortuosity is visually evident in the SEM image (Figure 2e) and can also be analyzed through pore size distribution measurements (Figure S2). The increased tortuosity is beneficial for blocking the growth of lithium dendrites. Combining high porosity (81.3%) and enhanced mechanical strength (25 MPa), we obtained an optimal balance among porosity for sufficient electrolyte uptake, adequate ionic conductivity for ion transport, structural rigidity for suppressing lithium dendrites, and network tortuosity for effectively regulating ion flux. As demonstrated in Figure S5, the Li^+^ transference numbers for the electrolyte with PP, electrospun PI, and SCPI separators are 0.46, 0.54, and 0.6, respectively. This higher Li^+^ transference number for SCPI separators can be attributed to the abundant polyimide framework in the SCPI separator; the polar functional groups in the polyimide can effectively interact with and immobilize the anions of the lithium salt,^28^ thereby facilitating the higher mobility for the lithium ions. Simultaneously, the excellent wettability of SCPI separators could further enhance the transportation of Li^+^ within the separator and promote uniform distribution of Li^+^ across the separator surface.^29^ The higher Li^+^ mobility indicates the smaller concentration polarization during cycling, which contributes to suppressing the formation and growth of lithium dendrites. A high anodic stability up to 4.71 V vs Li/Li^+^, confirmed by linear sweep voltammetry (LSV, Figure 4a,b), enables the SCPI separator’s application with high-voltage cathodes.

**Figure 4:**
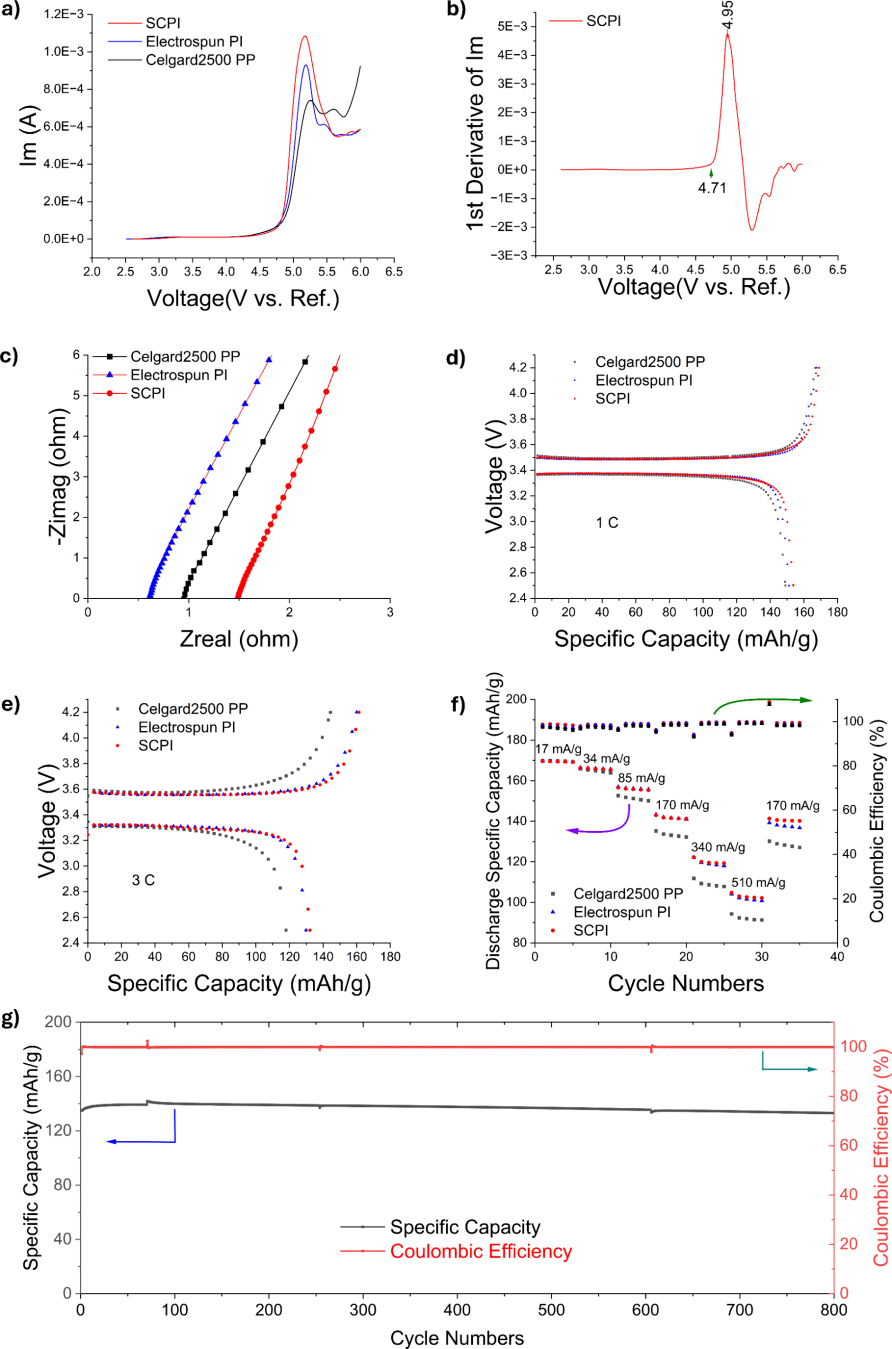
Electrochemical performances of Celgard2500 PP, electrospun PI, and SCPI separators. a,b) Electrochemical stability of the separators evaluated by linear sweep voltammetry (LSV). a: Comparison of the LSV curves for Celgard 2500 PP, electrospun PI, and SCPI separators. b: The first derivative of the LSV curve for the SCPI separator, identifying the onset of oxidation at 4.71 V vs Li/Li^+^. c) Nyquist plots of EIS for SS/separator/SS cells. d) Initial charge/discharge curves of Li/separator/LFP cells at 1 C (the test condition was set based on the nominal capacity of 160 mAh/g). e) Initial charge/discharge curves of Li/separator/LFP cells at 3 C (the test condition was set based on the nominal capacity of 150 mAh/g). f) Rate performances of Li/separator/LFP cells (the test condition was set based on the nominal capacity of 170 mAh/g). g) Long-term cycling performances of full pouch cells with graphite anodes, SCPI separators, and LFP cathodes at 1 C rate.

A key advantage of our PI-based separators is their ability to suppress the lithium dendrite growth. This was evaluated in Li/Li symmetric cells cycled at a demanding current density of 5 mA/cm^2^ with a capacity of 5 mAh/cm^2^. After 100 cycles, the lithium electrode from the cell with the PP separator was covered in large, mossy dendrite structures (Figure 3c), attributed to nonuniform current distribution caused by the separator’s irregular slit-like pores (Figure S4).^30,31^ In stark contrast, the lithium electrodes from cells using both the electrospun PI and SCPI separators remained remarkably smooth and flat, showing no evidence of dendrite formation (Figure 3d,e, where the left-to-right images show increasing magnifications of the same region).

This dendrite suppression and stable interface are directly reflected in the battery’s rate capability and cycling stability (Figure 4f,g and Figure S6). In Li/LFP coin cells, the discharge specific capacity for the SCPI separator consistently outperformed that for the PP separator at high rates, as evidenced by the smaller voltage gaps in the charge–discharge curves (Figure 4d,e). The practical viability of the SCPI separator was ultimately validated in a full pouch cell. As illustrated in Figure 4g, the pouch cell featuring SCPI separators demonstrated extraordinarily stable performance during long-term cycling. After 800 cycles at a 1 C rate, the cell retained 98.75% of its initial discharge capacity with an average Coulombic efficiency exceeding 99.99%. This remarkable stability provides compelling evidence that the SCPI separator’s unique structure effectively mitigates parasitic side reactions and enables long-life, high-performance batteries. The multiple characteristics of SCPI separators, such as superior pore structure, excellent wettability, improved mechanical strength, and increased Li^+^ transference number, could collectively contribute to the stability of prolonged cycling. An in-depth interfacial analysis, focusing on the chemical compositions and physical phases of the solid electrolyte interphase (SEI) and cathode electrolyte interphase (CEI), may further elucidate the underlying stability mechanism. Currently, cycling tests of the pouch cells are ongoing. Upon reaching 5000 cycles, the pouch cells are disassembled and a dedicated follow-up study will be conducted.

## 4 Conclusion

In this study, we successfully developed a novel self-composite polyimide (SCPI) separator by synergistically combining electrospinning and non-solvent-induced phase separation (NIPS). This approach addresses the critical weaknesses of conventional electrospun PI separators—namely, low mechanical strength and large, potentially unsafe pore sizes. The resulting single-material composite features a hierarchical structure, where a PI nanofiber scaffold is internally reinforced by a NIPS-derived PI matrix. This design yielded a separator with a 230% increase in tensile strength and a well-defined nanoporous network (200–500 nm) while retaining the outstanding thermal stability and electrolyte wettability inherent to polyimide.

Crucially, the uniform nanoporous structure and superior wettability have been shown to synergistically homogenize the Li-ion flux, resulting in highly effective suppression of lithium dendrite growth, as evidenced by the smooth Li metal surface after 100 cycles at 5 mA/cm^2^. This translated into exceptional long-term cycling stability in a practical pouch cell, which maintained 98.75% capacity retention after 800 cycles at 1 C. This work demonstrates that the “self-composite” strategy is a straightforward yet powerful method for engineering advanced membrane architectures, offering a viable pathway to developing the safe, reliable, and high-performance separators required for next-generation lithium batteries.

## Supplementary Material



## Notes

SEM images of surface morphologies; pore size distribution; wetting behaviors; morphologies of Celgard2500 PP separators; potentiostatic polarization and electrochemical impedance spectra; galvanostatic cycling tests (PDF)
