# Supplementary material for: Robust Self-Composite Polyimide Separators for Long-Life Lithium-Ion Batteries
Source: ACS Omega. 2026 Apr 14;11(16):24478–85. doi: 10.1021/acsomega.6c00560 (PMC13129815; doi:10.1021/acsomega.6c00560)
Supplement: Supplementary file 1 [file ao6c00560_si_001.pdf]

Supporting Information

Robust Self-Composite Polyimide Separators for Long-Life Lithium-Ion Batteries

*Shilong Bai[1] and Ping Gao[*1,2]*

[1]Advanced Materials Thrust, Function Hub, The Hong Kong University of Science and Technology (Guangzhou), Nansha District, Guangzhou, 511453, China. Email: kepgao@hkust-gz.edu.cn

[2]Department of Chemical and Biological Engineering, The Hong Kong University of Science and Technology, Clear Water Bay, HONG KONG SAR, PR China

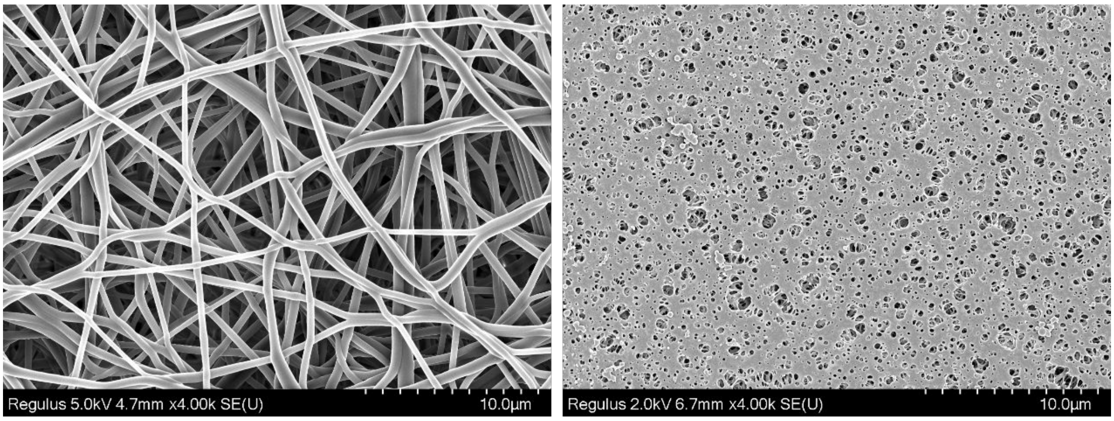

Figure S1 SEM Images of surface morphologies. Left: Electrospun PI separators; Right: SCPI separators. Scale bar: 10 μm.

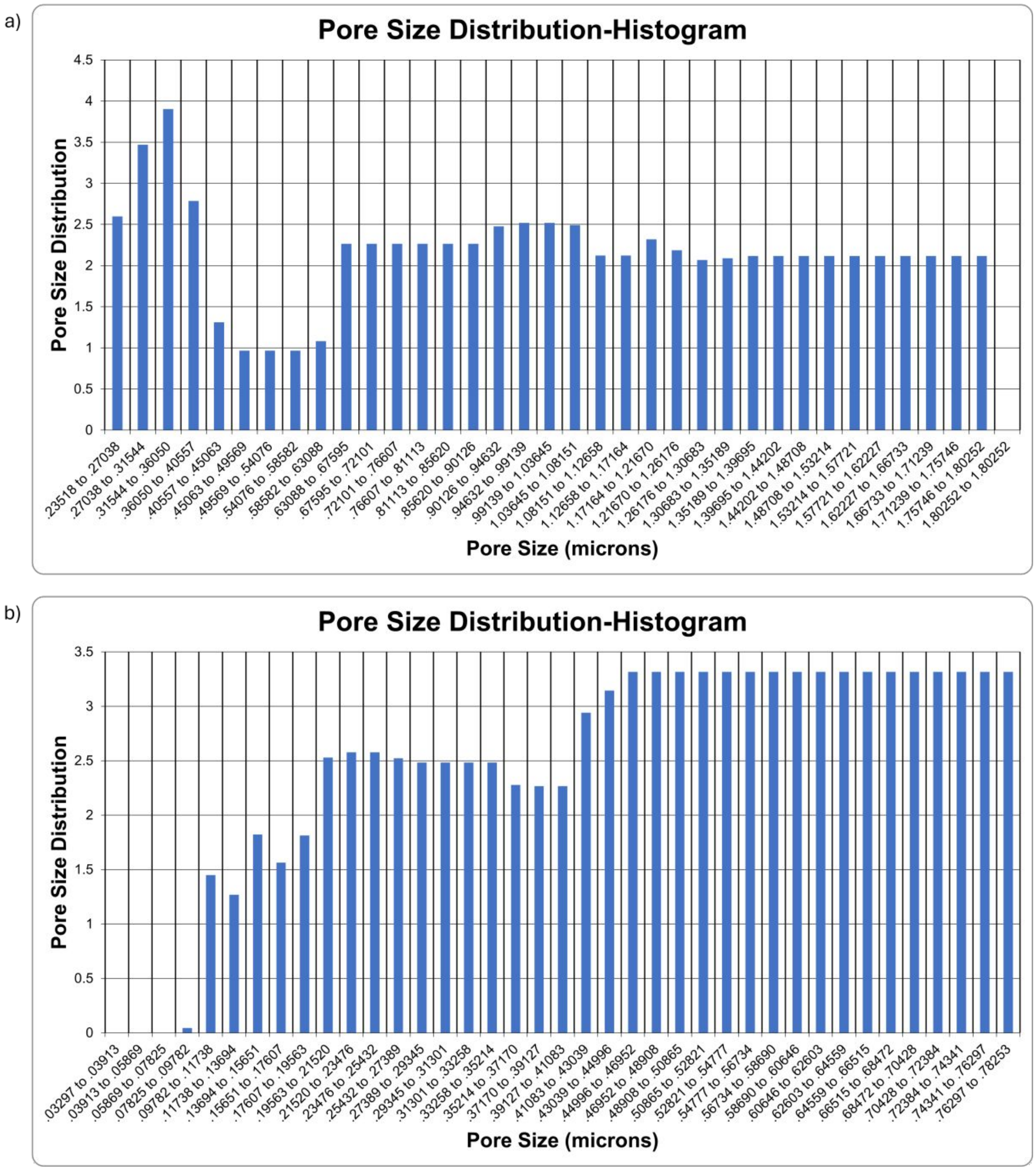

Figure S2 Pore size distribution. a) Electrospun PI separator. b) SCPI separator.

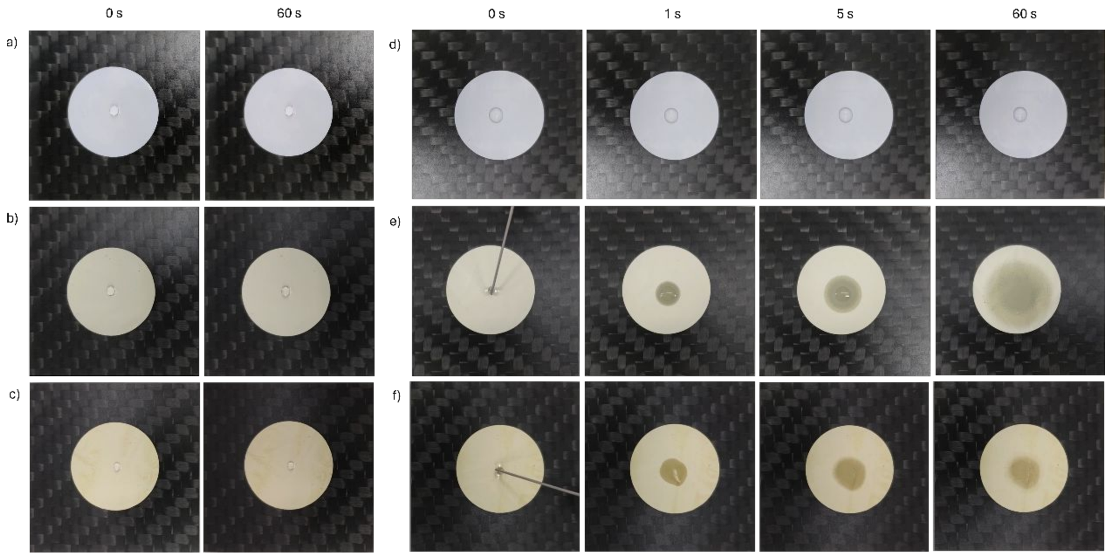

Figure S3 Wetting behaviors. a) Water on Celgard2500 PP separators. b) Water on Electrospun PI separators. c) Water on SCPI separators. d) PC on Celgard2500 PP separators. e) PC on Electrospun PI separators. f) PC on SCPI separators.

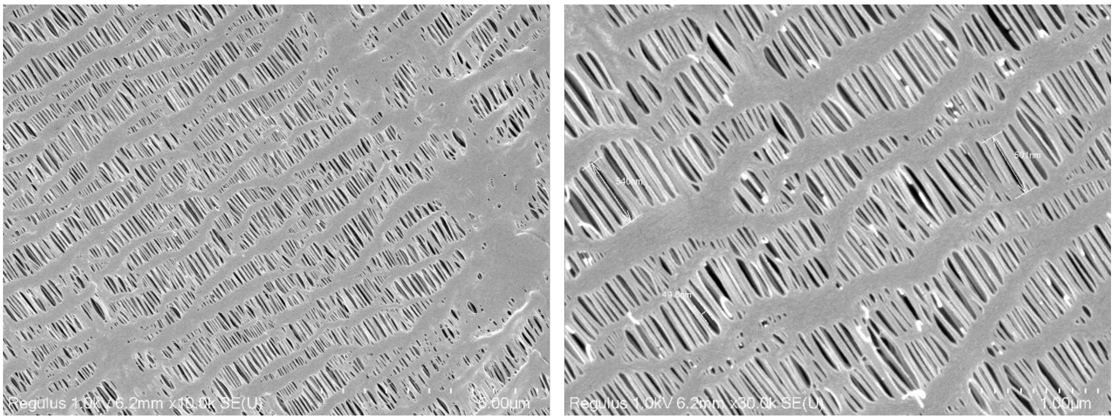

Figure S4 Morphologies of Celgard2500 PP separators. Scale bar: 5 μm (Left); 1 μm (Right).

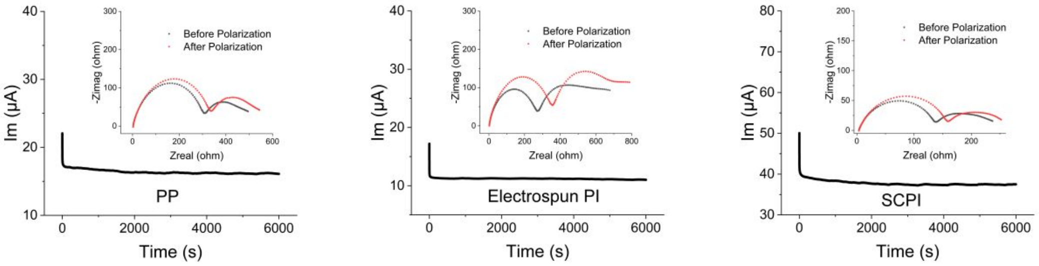

Figure S5 Potentiostatic polarization and electrochemical impedance spectra of Celgard2500 PP, Electrospun PI and SCPI separators. Left: Celgard2500 PP; Middle: Electrospun PI; Right: SCPI.

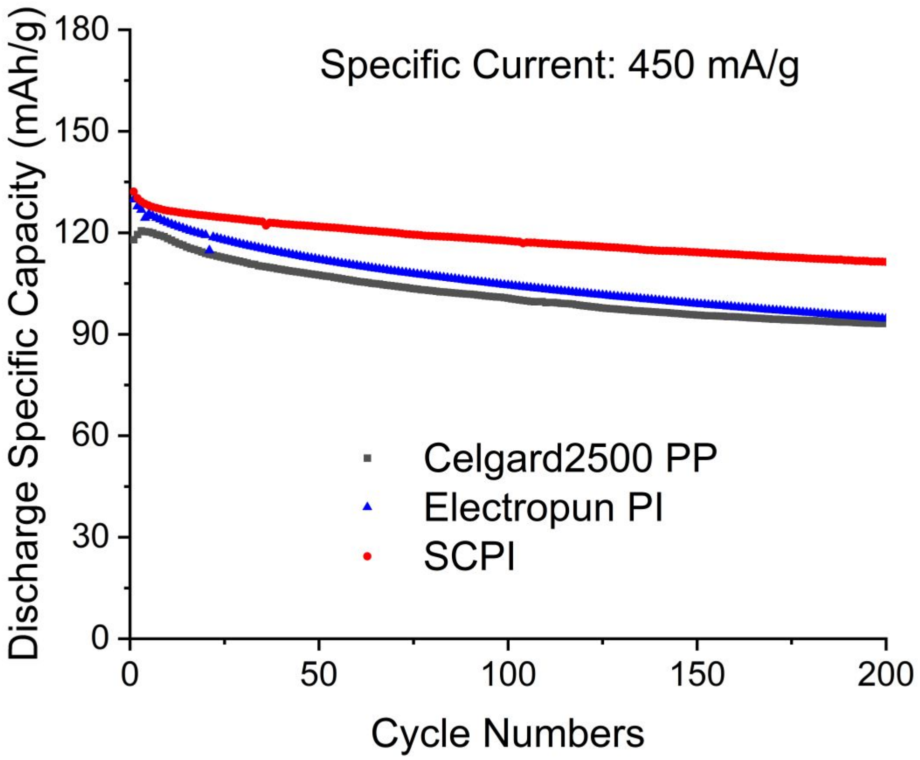

Figure S6 Galvanostatic cycling tests of Li/separator/LFP cells using SCPI, electrospun PI, and Celgard2500 PP separators at 450 mA/g (~3 C rate).
